# Does pre-ordering tests enhance the value of the periodic examination? Study Design - Process implementation with retrospective chart review

**DOI:** 10.1186/1472-6963-11-216

**Published:** 2011-09-13

**Authors:** Vicki L Hunt, Rajeev Chaudhry, Robert J Stroebel, Frederick North

**Affiliations:** 1 Mayo Clinic, Division of Primary Care Internal Medicine, 200 1st Street SW, Rochester, MN 55905, USA

## Abstract

**Background:**

To evaluate the value of a pre-ordering process for the pro-active scheduling and completion of appropriate preventive and chronic disease monitoring tests prior to a periodic health examination (PHE).

**Methods:**

A standardized template was developed and used by our nursing staff to identify and schedule appropriate tests prior to the patients PHE. Chart reviews were completed on all 602 PHE visits for a 3-month interval in a primary care setting. A patient satisfaction survey was administered to a convenience sample of the PHE patients.

**Results:**

Of all the patients with tests pre-ordered, 87.8% completed the tests. All providers in the division used the process, but some evolved from one template to another over time. Most patients (61%) preferred to get their tests done prior to their PHE appointment. Many of our patients had abnormal test results. With this process, patients were able to benefit from face-to-face discussion of these results directly with their provider.

**Conclusions:**

A pre-order process was successfully implemented to improve the value of the PHE visit in an internal medicine primary care practice using a standardized approach that allowed for provider autonomy. The process was accepted by patients and providers and resulted in improved office efficiency through reduced message handling. Completion of routine tests before the PHE office visit can help facilitate face-to-face discussions about abnormal results and subsequent management that otherwise may only occur by telephone.

## Background

Patients clearly benefit from preventive services. Some preventive services, such as immunization, can reduce disease risk. Other preventive services, such as cancer screenings, can provide early detection and curative treatment. The success of preventive services in improving the health of patients has made the delivery of these services a top priority of healthcare in the US. Preventive services were prominently displayed as examples of underused services by the Institute of Medicine's (IOM) landmark book "Crossing the Quality Chasm"[1] The IOM challenged the nation to improve the delivery of preventive service. A dilemma medical practices face is how to deliver the myriad of recommended preventive services efficiently. It is clear that physicians lack the time to do it all. Studies estimate that the time required to deliver all the recommended preventive services to a normal panel size of 2,500, ranges from 8 to 22 hours daily [2,3]. A systems approach to this problem has been partially successful [4]. At Mayo Clinic's campus in Rochester, MN systems approaches to mammography screening and osteoporosis screening have resulted in significant improvements in care delivery to patients without requiring physician contact [5,6]. Other computerized reminder systems help increase attention to preventive services at the point of care [7].

Despite some success of automated systems to improve the delivery of preventive services, there is sufficient evidence to suggest that providers should still be playing an active role in the process. In addition to the evaluation of underlying concerns and chronic disease management, the PHE also identifies and rectifies gaps in recommended preventive services. The PHE is intended to provide the time necessary for education, counseling, and ordering of these tests. A recent systematic review on the PHE came to the conclusion that it improved the delivery of some recommended preventive services [8]. Another recent study confirmed that the PHE improves delivery of cancer screening [9].

The standard practice of identifying and ordering screening and chronic disease monitoring tests at the PHE office visit is not satisfying or efficient. Valuable provider time is expended identifying and ordering tests. Prior to implementation of the PHE pre-order process, the standard practice was for the provider to identify and order preventive services at the time of the PHE. To improve appointment capacity for necessary face-to-face visits, the division eliminated routine return visits for test results in 2002. Patients are now notified of routine test results generated from a PHE visit via telephone calls from the provider or nursing staff, or by a letter sent to them in the mail. On a previous internal audit at the Mayo Clinic, we determined that call backs for test results can be extremely time consuming, requiring an average of 7.5 messages back and forth between the nurse, provider, and patient, before patients are informed of the result. Likewise, a letter takes several minutes to complete and has additional stationary and postal expense. Patients also have strong preferences for how they like to receive test results, and they prefer their own doctor inform them of their results [10].

We wanted to improve the efficiency of the PHE process by implementing a system to pro-actively identify, order, and schedule appropriate services prior to a PHE. Our aim was to improve the value of the PHE by ordering and obtaining preventive and chronic disease monitoring tests prior to the PHE so the results would be available for discussion at the time of the office visit. We also wanted to assess the acceptability of this process to patients who would get their testing completed at a different time from their PHE appointment, often requiring an extra visit. Herein, we recount our experience with development and implementation of the process to accomplish these aims.

## Methods

The study took place in the Division of Primary Care Internal Medicine (PCIM) at Mayo Clinic, Rochester, Minnesota. At the time of the study, there were 41 internists in PCIM who were split into smaller care teams staffed with 5 to 6 physicians, 1 Nurse Practitioner or Physician's Assistant, 2 to 3 patient appointment coordinators (PAC), 3 licensed practical nurses (LPN), and 3 registered nurses (RN). The division serves 42,000 patients and managed over 88,000 office visits in 2009. All patients served by the division are age fifteen or older. This study was reviewed by the Mayo Clinic Institutional Review Board (Application number- 10-000383). The Board determined the study involved a quality assurance project and did not constitute research involving human subjects.

**Figure 1:**
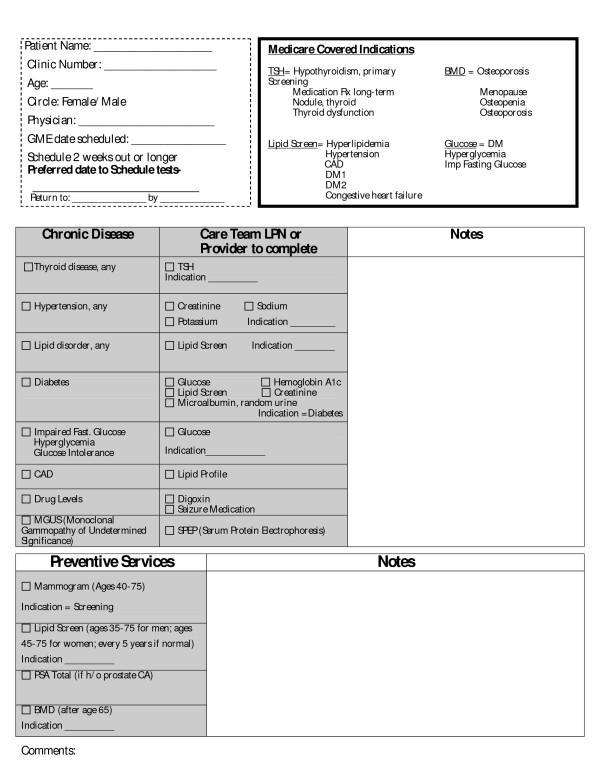
**Pre-orders template used by allied health staff to determine what tests to order for a patient before a PHE visit**.

Most appointments for a PHE are requested by the patient who desires a comprehensive medical evaluation. Some appointments are requested by the primary care provider for patients with chronic diseases that would benefit from a comprehensive PHE. The pre-orders process was limited to PHE appointments, which were scheduled at least 2 weeks in advance. We created a standard template that outlined preventive service recommendations based on the Institute for Clinical Systems Improvement (ICSI) guidelines (Figure 1).(ISCI website; http://www.icsi.org) Providers were allowed to modify the standard template and create a provider specific template to fit individual practice styles for additional testing as long as established guidelines continued to be followed for routine services. In each of the care teams, a licensed practical nurse (LPN) was trained to use the template to identify and order indicated preventive services tests before a scheduled PHE. PAC staff was trained to offer pre-ordering when a patient called by telephone to schedule a PHE visit and the electronic record indicated services were due. Training was provided to LPN staff about evidence based preventive screening and chronic disease monitoring consistent with ICSI guidelines. Several mock patients were reviewed with provider and LPN staff to assist in LPN training and assure accurate ordering. Prior to the PHE visit, the nurse would review the patient's medical record and order the indicated tests. With the standardized template, the nurse examined the record for specific chronic diseases (hypertension, diabetes, hyperlipidemia, etc.) and the ordering of standard tests to follow these conditions was done by protocol. The standard template also contained protocols for ordering cancer related screening. Customized templates had minor individual provider variations to the standard. For example, some providers wanted AST or ALT levels for all their patients getting lipid panels. Others wanted fasting glucose levels on all patients, and some wanted mammograms continued beyond age 75. All lab tests were scheduled to be completed within 7 days of the scheduled office visit. We termed this the PHE pre-order process.

Each provider was given basic instructions on how the process worked and how the LPN could pre-order tests prior to a scheduled PHE. For each provider, the pre-orders process was strictly voluntary. Providers were free to decline the pre-orders process for their PHE appointments from the very start or stop using it at any time. Providers had the option of using either the standard or a customized template and could either complete the template themselves or have the LPN complete it. Providers also had the option to review the templates once they were completed by the LPN.

We collected data from 602 pre-orders templates initiated from June 15, 2009 through July 30, 2009 for patients who had requested a PHE. Chart review was performed for all 602 of those patients. Data collected from the pre-order template was the clinic number, date of template initiation, provider name, LPN name, and number of tests ordered prior to the PHE. For the purposes of the study, a test orderable as a bundled item was counted as one test. For example, a lipid panel was counted as one test, even though it included triglycerides, HDL, LDL, and total cholesterol values. A test was considered abnormal if the laboratory flagged the result as such. Data collected from chart review was the date of the PHE, and number of tests ordered on the date of the PHE.

A convenience sample of 44 office visit patients to PCIM were asked about their preferences for PHE testing.

The 7 LPNs who used the pre-orders templates identified the provider's use of the preorders templates (standard vs. custom) in 2008 and how they were using it in 2009 at the time of the study. The nurses also tracked the time spent to complete the preorders tasks of chart review and ordering tests.

We used JMP 8.01 (SAS Institute, Cary, NC) for the descriptive statistics and data analysis. The T-test was used to calculate the p value for the difference in means of the number of tests ordered.

## Results

Females accounted for 374 (62%) of the pre-orders. The mean age for all patients with pre-orders was 61.8 years (CI 95% = 60.5 to 63.0%).

All providers in the division used the process (41/41). At initiation of the study, the standard template was used by 11 (26.8%) of the providers, while the other 30 (73.2%) providers elected to add specific orders to the standard template, therefore, establishing a customized template to correspond to their individual practice styles.

Nursing time to do the pre-orders depended on the degree of involvement of the provider in the process. For those providers who allowed the LPN to accomplish the preorders process independently (without direct provider input), it took the LPN 6.78 minutes (CI 95% = 6.38 to 7.19, N = 356) to review the patients chart and issue indicated orders. For those providers who desired more direct input into the process it took the LPN 4.44 minutes (CI 95% = 3.64 to 5.24, N = 97) to review a patients chart and prepare the appropriate template for the provider review. The time difference was due to the LPN not placing orders before provider review.

**Figure 2:**
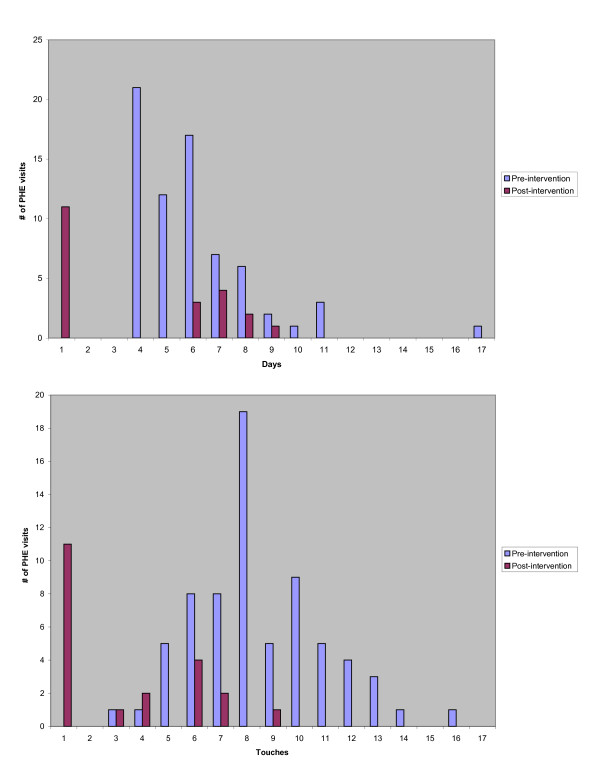
**Pre-intervention and post-intervention distribution of the number of days until patient received test results (top) and number of times a message related to a PHE test result was handled by care team staff (bottom)**.

An earlier pilot of this process resulted in a 69% reduction in message handling and a 40% reduction in the days it took to communicate test results to the patient. (Figure 2)

**Figure 3:**
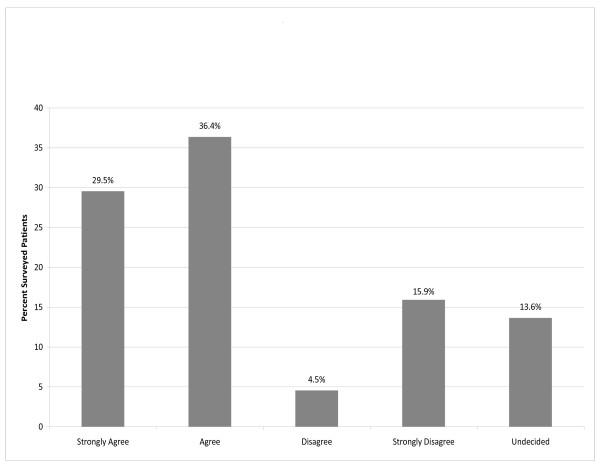
**Patient survey answer to question "It is important to me that before my examination, I have appropriate tests ahead of time so results are available for discussion face-to-face with my provider**."

The patient survey showed a majority of patients preferred getting studies completed before the PHE. For the question "Do you prefer to have tests completed before your appointment?", 27 patients answered "yes" (61%, CI 95% = 47% to 74%), while 15 (34%, CI 95% = 22% to 49%) answered "don't care". A 5-category Likert scale question "It is important to me that, before my examination, I have appropriate tests completed ahead of time so results are available for discussion face-to-face with my provider", resulted in "strongly agree" or "agree" for 29 patients (65.9%, CI 95%= 51% to 80%). (Figure 3)

**Figure 4:**
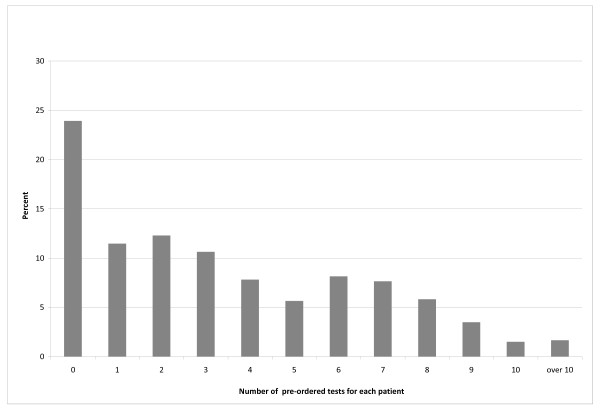
**Distribution of the number of tests ordered for each patient by the pre-orders process**.

Of the 602 patients who had an appointment for a PHE, with the pre-orders process, 590 (98%) attended the appointment. A total of 2082 total tests were preordered for these patients. Tests were pre-ordered for 76.1% (CI 95% = 75.5% to 79.3%) of these patients. Nursing pre-ordered a mean number of 3.46 (CI 95%= 3.7 to 3.2) tests per patient (Figure 4). Of all the patients with tests pre-ordered, 87.8% (CI = 84.5-90.5) completed the tests.

Of the 602 total patients that had a periodic health maintenance visit scheduled, 277 had abnormal lab results (46%). The mean number of abnormal lab results for each patient was 0.86 (CI 95% = .77 to.95). Of the 458 patients that had pre-ordered labs, 277 had at least one abnormal lab results (61.0%; CI 95% = 56.4 to 65.4). For this 61%, this provided an opportunity for a face-to-face discussion of these abnormal results with provider. There were a total of 509 abnormal results out of 458 patients.

Of the patients that attended the appointment, 71% had additional tests or consults ordered on the day of the visit. There were 196 patients (33.2%; CI 29.5 to 37.1) who had consultations ordered and, 1419 total tests were ordered on the day of the office visit out of a total of 590 patients that attended the appointment. Additional blood tests ordered at the office visit can often be done with stored serum, therefore, eliminating the need for another venipuncture. Our laboratory stores serum for 7 days after a blood draw. A mean of 2.4 (CI = 2.67-2.13) total additional orders were issued per patient by the provider on the day of the visit. Patients with no tests pre-ordered (N = 144) had significantly more tests ordered at the time of examination (mean = 3.08, 95%CI 2.62 to 3.55) than those who had tests preordered (mean tests ordered examination day = 2.20, 95% CI 1.94 to 2.46). This represented a 0.88 absolute increase in number of tests ordered for the usual care group compared to the pre-orders group (p = 0.001) and represented a 40% relative increase over the number of tests ordered by the pre-orders group.

## Discussion

In this study, we were able to demonstrate a pre-orders process that was responsive to the desires of patients, universally acceptable to physicians, sustainable, and only took a few minutes of time to accomplish by ancillary staff. To our knowledge, this is the first study to quantify the impact of such a process on test ordering before and after the PHE and the time cost involved.

The pre-order process is both a patient and provider satisfier. It should be noted that this process was completely voluntary for all patients and providers. The acceptance rate was close to 90% for patients and 100% for providers. The high acceptance rate by patients is consistent with our satisfaction survey which showed the majority of patients preferred to have tests ordered in advance of their periodic health examination. Coming in for tests in advance of the PHE may have posed some inconvenience for some patients. However, the high patient compliance, demonstrated by the lab completion rate of 87.8% suggests that they perceive this process as added value to their PHE visit. Although we did not perform a provider satisfaction survey, the 100% voluntary participation by providers, which has been sustained, speaks for itself as an indicator of provider satisfaction with the process.

Providers were given a large degree of autonomy to modify the pre-orders template. We feel that the autonomy was important to achieve such high provider buy-in. When the process was initially rolled out as a single standardized template, it quickly became apparent that providers desired the flexibility to customize the template to allow for minor differences in practice style. Once the process was adapted to allow providers the autonomy to make minor changes to the template, all providers embraced the process and have continued to do so.

Our data suggests some reasons as to why the pre-orders acceptance rate is high among patients and providers. A large percentage of patients had abnormal test results. With this process, patients are able to benefit from face-to-face discussion of results directly with their provider. Previously, most patients would have received test results by telephone from a nurse. This would often require additional information for the patient from the provider. We know from a previous study in our patient population that patients prefer to receive tests results directly from their provider [10]. One would expect this to be even more important when tests results are abnormal. A face-to-face discussion also allows for development of a patient-centered plan to manage results of abnormal tests.

Although this process did not eliminate the need for additional orders at the time of the PHE, 29% of the patients required no further tests ordered. The provider has more time to focus discussion on specific patient concerns instead of ordering routine tests. The patient is able to be involved in his own care. This process is very much in line with how a patient centered medical home should function. The provider has more time during the PHE to be perceived as the coordinator of care, rather than merely reporting results by telephone at a later time.

The importance of addressing the patient face-to-face with abnormal results and when ordering consultations should not be underestimated with this process. Not only does the patient have the opportunity to question the doctor about further tests and consultations, the doctor in turn, is able to fulfill the role expected of them in the medical home model by visibly showing the patient their active role in coordinating care. Our process also demonstrates patient centric team based care coordination prior to the office visit. Even before the patient comes to the PHE, the patient is aware that tests are ordered customized to their particular needs, further establishing the medical home model in the mind of the patient.

Although this process could be generalized to other practices, it is possible that patient acceptance may not be as high. For example, it may be more inconvenient to young, healthy patients that are more likely to have normal test results. For those with normal test results, the value added by face-to-face discussion would likely be less. For those populations having mostly normal results, it may be more acceptable to have tests done on the same day after the PHE, with the normal results reported by telephone or by electronic means. Further study is needed to determine whether the pre-orders process would be as desirable in other population groups.

This study had limitations. Our only control group is historical and the number of face-to-face discussions about PHE tests were quantifiable and was zero unless the provider scheduled an additional visit. Telephone calls for test results were not measured for the intervention or control group. We did not fully analyze the downstream cost of the pre-orders process. However, we think that the 4-to-7 minute time taken by the LPN to complete the pre-orders process pays for itself by a decrease in downstream rework done both by the provider and the nurse. A future study could look specifically at the start to finish work required for the pre-orders process compared to ordering at the time of the PHE. Although we are able to add many laboratory tests to stored serum, these tests must be run within 7 days of the original blood draw. Therefore, not all tests can be run on stored serum.

## Conclusion

In conclusion, we successfully implemented a pre-order process to improve the quality of the PHE visit in an internal medicine primary care practice using a standardized approach allowing for provider autonomy. It was readily implemented by LPNs with minimal training and was accepted by patients and providers. The pre orders process creates more face-to-face opportunity for the provider to engage in patient centric activity which is necessary in the medical home model.

## Abbreviations

IOM: Institute of Medicine; LPN: Licensed Practical Nurse; PAC: Patient Appointment Coordinator; PHE: Periodic Health Examination; RN: Registered Nurse.

## Competing interests

The authors declare that they have no competing interests.

## Authors' contributions

VLH participated in the design of the study, data collection, and drafting of the manuscript. FN participated in the design of the study, data collection and analysis, and drafting of the manuscript. RC and RJS critically reviewed the manuscript. All authors read and approved the final manuscript.

## Pre-publication history

The pre-publication history for this paper can be accessed here:

http://www.biomedcentral.com/1472-6963/11/216/prepub
